# Association of Educational Attainment With Adiposity, Type 2 Diabetes, and Coronary Artery Diseases: A Mendelian Randomization Study

**DOI:** 10.3389/fpubh.2020.00112

**Published:** 2020-04-22

**Authors:** Min Cao, Bin Cui

**Affiliations:** Shanghai Clinical Center for Endocrine and Metabolic Diseases, Shanghai Institute of Endocrine and Metabolic Diseases, Ruijin Hospital, Shanghai JiaoTong University School of Medicine, Shanghai, China

**Keywords:** Mendelian randomization, educational attainment, adiposity, type 2 diabetes, coronary artery disease

## Abstract

**Background:** Observational studies showed that educational attainment (EA) is associated with cardiometabolic diseases, but the long interval between exposure and outcome makes it difficult to infer causality. We herein used Mendelian randomization (MR) to examine the causal effects of EA on adiposity, type 2 diabetes (T2D), and coronary artery disease (CAD).

**Methods:** A two-sample MR analysis was conducted using genome-wide association study (GWAS) summary statistics. Seventy-four instrumental variables (IVs) were used to determine the causal effect of EA on cardiometabolic diseases. Sensitivity analyses were also performed to detect the pleiotropy of the IVs.

**Results:** Using the MR approach, we found that each additional year in EA is associated with a reduction in the body mass index (BMI) (β −0.17 [95% CI −0.23, −0.10], *P* = 8.85 × 10^−7^), a 39% reduction in the odds of having T2D (OR 0.61 [95% CI 0.50, 0.75], *P* = 1.16 × 10^−6^), and a 36% reduction in the odds of having CAD (OR 0.64 [95% CI 0.55, 0.75], *P* = 2.38 × 10^−8^) at the Bonferroni-adjusted level of significance.

**Conclusion:** Our findings suggest a causal role of EA on the cardiometabolic diseases including adiposity, T2D, and CAD.

## Introduction

Observational studies have consistently showed that socioeconomic status such as low education is associated with an increased risk of cardiovascular disease (CVD) and mortality (1–4), but disentangling causality is challenging due to the long interval between exposure and outcome. Mendelian randomization (MR) analysis may help to clarify the relationship (5), which utilizes genetic variants as the unconfounded proxies for an exposure of interest and leverages the random assortment of alleles at the time of conception to overcome limitations inherent in observational studies, thus improving causal inferences. The MR method has been widely used in a range of biological and behavioral exposures, but very few in socioeconomic exposure. Two MR analyses revealed the causal role of educational attainment (EA) on myopia (6) and obesity (7), but both using relatively small sample size. A recent MR study indicated a causal association between low EA and increased risk of smoking, which might explain the observational associations between EA and adverse health outcomes such as coronary artery disease (CAD) (8). Relatedly, a well-designed MR study found that additional education was causally associated with a decreased risk of CAD, and proposed that smoking, body mass index (BMI), and blood lipids might be the potential mechanisms, but no causal effect was found on type 2 diabetes (T2D) (9). The recent findings in an MR study suggested that a large part of the protective effect of education on CVD was mediated by BMI, systolic blood pressure, and smoking behavior (10).

In this study, in order to determine the causal effect of EA on adiposity, T2D, and CAD, we used a two-sample MR approach obtaining publicly available summary statistics from genome-wide association studies (GWAS) of EA (11) and the outcomes (12–14). We also tested the robustness of our findings across a range of MR methods as sensitivity analyses.

## Methods

### Study Design

We performed two-sample MR studies based on the publicly available summary-level data from GWASs to determine the causal relationship between EA and BMI, T2D, and CAD. The genetic instrumental variables (IVs) applied in the MR analysis must satisfy the following three assumptions (15): (1) the genetic variants used as IVs must be associated with EA, (2) the genetic variants must not be associated with any confounders, and (3) the genetic variants must influence BMI, T2D, and CAD only through EA but not through any direct or alternative pathways.

### IV Selection and Data Sources

Summary statistics of EA associated single nucleotide polymorphisms (SNPs) were extracted from a GWAS incorporating 293,723 individuals of European descent, and a replication cohort of 111,349 individuals from the UK Biobank (11). Throughout all analyses, we defined education in the same way as in the original GWAS analysis, in which data from 65 studies were harmonized against the International Standard Classification of Education 1997 classification system (11). A total of 74 SNPs were reported as being associated with EA at the genome-wide significance level (*P* < 5 × 10^−8^) (11). To ensure that all the IVs are not in linkage disequilibrium (LD) with each other, we assessed correlation LD between all the selected SNPs in the European subset of 1,000 Genomes (phase 3) via LDlink (16). When the correlation coefficient between SNPs was high (*r*^2^ > 0.05), we discarded the SNP with the larger *P* value (17). A total of six SNPs (rs13402908, rs4500960, rs4851251, rs2245901, rs148734725, and rs324886) were removed after LD assessment and 68 SNPs were left as IVs for further analysis.

Summary-level data were extracted from the Genetic Investigation of ANthropometric Traits (GIANT) consortium (*n* = 322,154) for the BMI (12), from the Diabetes Genetics Replication and Meta-analysis (DIAGRAM) consortium (*n* = 159,208) for T2D (13), and from the Coronary Artery Disease Genome-wide Replication and Meta-analysis (CARDIoGRAM) plus the Coronary Artery Disease (C4D) Genetics (CARDIoGRAMplusC4D) consortium (*n* = 184,305) for CAD (14). The BMI (measured or self-reported weight in kilogram per height in meters squared), a measure commonly used to assess adiposity, was adjusted for age, age squared, and any necessary study-specific covariates in a linear regression model (12). Case subjects with T2D were diagnosed according to the 1999 World Health Organization criteria of fasting plasma glucose concentration ≥ 7.0 mmol/L or 2-h plasma glucose concentration ≥ 11.1 mmol/L, by a report of diabetes medication use, or based on medical record review (18). CAD was determined with a broad definition including myocardial infarction, acute coronary syndrome, chronic stable angina, or coronary artery stenosis > 50% (14). When target SNPs were not available in the outcome study, we used proxy SNPs that were in high LD (*r*^2^ > 0.8) with the SNPs of interest. Two variants (rs1871109 and rs1606974) were discarded in the analyses for T2D and CAD as no suitable proxy SNP was found (Supplementary Table 1), and another 10 (rs10061788, rs112634398, rs12646808, rs12772375, rs165633, rs17824247, rs2610986, rs35761247, rs62259535, and rs8005528) were removed in the analysis for adiposity (Supplementary Table 2).

Ethical review and informed consent had been obtained in all of the original studies.

### Statistical Analysis

The estimates of the causal effect of EA on the outcomes were analyzed using the inverse variance–weighted (IVW) method, median-based method, and MR-Egger for multiple genetic variants. The IVW method was used to provide a combined estimate of the causal estimate from each SNP (19). Median-based methods, including the simple median-based method and the weighted median-based method, have greater robustness to individual genetics with strongly outlying causal estimates compared with IVW and MR-Egger methods, which give a consistent estimate of the causal effect when at least 50% of the genetic variants are valid IVs (20). The MR-Egger regression test was used to evaluate the directional pleiotropy and investigate the null causal hypothesis under the InSIDE (Instrument Strength Independent of Direct Effect) assumption (15). In this study, the MR-Egger regression (15) and the Cochran's *Q* test (21) were used as sensitivity analyses to investigate the presence of pleiotropy. All statistical analyses were performed using R v3.5.3 (the R Foundation) and the related package (*Mendelian Randomization*) (22). The Bonferroni-adjusted level of statistical significance for the BMI, T2D, and CAD was *P* < 0.017 (0.05/3 = 0.017) to account for three tests.

## Results

### Education and BMI

A total of 56 IVs were used in the MR analysis after LD assessment and data availability check. Standard IVW MR analysis showed that 1 year longer education was associated with a reduction in the BMI (β −0.17 [95% CI −0.23, −0.10], *P* = 8.85 × 10^−7^) at the Bonferroni adjusted level of significance (*P* < 0.017; Table 1). The intercept term estimated for the BMI from MR-Egger regression did not differ from 0 (intercept estimate −0.003, 95% CI −0.001 to 0.004, *P* = 0.34), suggesting that individual SNP heterogeneity was largely balanced (Table 1).

**Table 1:** MR analyses of the causal effect of EA on BMI, T2D, and CAD.

**Traits**	**SNPs**	**Simple median-based method**		**Weighted median-based method**		**IVW method**		**MR-Egger regression**				**Cochran's Q test**
		**β (95% CI)**	***P*-value**	**β (95% CI)**	***P-*value**	**β (95% CI)**	***P*-value**	**Intercept β (95% CI)**	***P*-value**	**MR-Egger β (95% CI)**	**MR-Egger *P*-value**	***P*-value**
BMI	56	−0.19 (−0.29, −0.08)	0.001	−0.22 (−0.32, −0.12)	3.44 × 10^−5^	−0.17 (−0.23, −0.10)	8.85 × 10^−7^	−0.003 (−0.010, 0.004)	0.34	0.04 (−0.39, 0.46)	0.87	1.49 × 10^−11^
T2D	66	−0.76 (−1.08, −0.44)	2.89 × 10^−6^	−0.67 (−0.99, −0.36)	2.99 × 10^−5^	−0.49 (−0.69, −0.29)	1.16 × 10^−6^	−0.014 (−0.032, 0.003)	0.11	0.32 (−0.73, 1.36)	0.55	3.92 × 10^−7^
CAD	66	−0.54 (−0.78, −0.30)	9.19 × 10^−6^	−0.44 (−0.67, −0.21)	2.11 × 10^−4^	−0.44 (−0.60, −0.29)	2.38 × 10^−8^	−0.005 (−0.016, 0.007)	0.44	−0.18 (−0.87, 0.50)	0.60	0.02

Furthermore, the simple median–based method and the weighted median–based method were also used to estimate the causal effect of EA on the BMI and further proved the significant effects on adiposity, supporting the robustness of our findings (Table 1).

### Education and T2D

**Figure 1:**
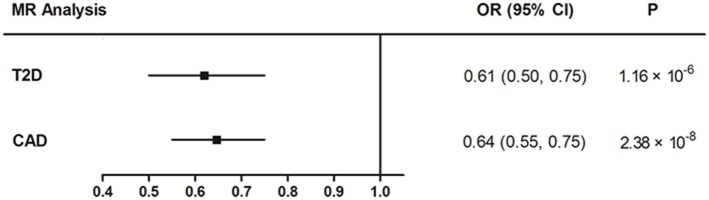
MR estimated effects of EA on T2D and CAD. OR, odds ratio; 95% CI, 95% confidence interval.

The IVW method using the 66 IVs demonstrated a causal effect of EA on T2D, with 1-year increase in EA causing a 39% reduction in the odds of having T2D (OR 0.61 [95% CI 0.50, 0.75], *P* = 1.16 × 10^−6^) (Figure 1). There was moderate evidence of heterogeneity to suggest the presence of horizontal pleiotropy, with a Cochran's Q test *P*-value of 3.92 × 10^−7^. MR-Egger regression analysis did not provide evidence of directional horizontal pleiotropy (intercept estimate −0.014, 95% CI −0.032 to 0.003, *P* = 0.11) (Table 1). The sensitivity analyses using the simple median–based method and the weighted median–based method also demonstrated similar causal effects, with additional EA associated with the reduction in the odds of having T2D (β −0.76 [95% CI −1.08, −0.44], *P* = 2.89 × 10^−6^ and β −0.67 [95% CI −0.99, −0.36], *P* = 2.99 × 10^−5^, respectively) (Table 1).

### Education and CAD

Similarly, the IVW MR result showed that each additional year in EA was associated with a 36% reduction in the odds of having CAD (OR 0.64 [95% CI 0.55, 0.75], *P* = 2.38 × 10^−8^) (Figure 1). The intercept term estimated from MR-Egger regression was centered at the origin with a CI including the null, showing that the observed results were not influenced by pleiotropy (Table 1). The simple median–based method and the weighted median–based method both showed consistent results with that of the IVW method (Table 1).

## Discussion

In the use of MR analysis, we found that longer EA was causally associated with the decreased odds of having cardiometabolic diseases, which is consistent with the traditional observational studies showing that socioeconomic status was associated with obesity, T2D, CVD, and even life expectancy (4, 23–27).

The MR method has been widely adopted to assess the causality. In the current study, we selected the commonly used education-associated SNPs as IVs and only kept the independent SNPs after LD pruning. The MR-Egger regression results showed that our findings were not being influenced by pleiotropy. Our findings may provide additional evidence for the causal role of EA on adiposity, T2D, and CAD since the influence of traditional confounding factors in observational studies is minimized. Limited evidence of MR analysis on education has been reported, especially for cardiometabolic diseases. An MR study showed a negative causal effect of education on the BMI using the same IVs as in our study; however, the sample size is too small (*N* = 2,011) (7). The recent findings used the MR method to demonstrate a strong inverse genetic correlation between EA and CAD risk, as well as the BMI, smoking, and blood pressure, but not T2D (9, 10), while our current study used the widely accepted IVs for EA and the updated summary data from GWAS reports to demonstrate the causal effect of EA on the BMI and CAD, and also revealed its significant association with T2D (*P* = 1.16 × 10^−6^). In addition, the sensitivity analyses, including IVW the method, the simple median–based method, and the weighted median–based method, all showed consistent causal effects, supporting the robustness of our findings.

Even for a behavioral phenotype like EA that is mostly environmentally determined, a well-powered GWAS identifies replicable associated genetic variants that suggest biologically relevant pathways. The IVs used in the study are disproportionately found in genomic regions regulating gene expression in the fetal brain, and the candidate genes are preferentially expressed in neural tissue, especially during the prenatal period, and enriched for biological pathways involved in neural development (11). Because EA is measured in large numbers of individuals, it will be useful as a proxy phenotype in efforts to characterize the genetic influences of related phenotypes, including cognition and neuropsychiatric disease. Moreover, cognition and brain function have been reported to be associated with cardiometabolic diseases (28–30).

Our study has several important strengths. First, the causal effect revealed by the MR method will not be influenced by the confounding factors, which is a major limitation of the observational studies. Second, using the large and updated GWAS summary data, our study could have sufficient power to assess the potential causal effects.

There are also some limitations in our study. First, the causal associations between EA and adiposity, T2D, and CAD were consistent in IVW and median-based methods, but the result from MR-Egger was less compelling, suggesting that IVW estimates may be biased by other confounding factors. However, we note that MR-Egger is generally considered as only one of the sensitivity analyses used to assess the validity of MR findings (20). Second, majority of the samples in our study were of European origin from high income countries, which would bring bias due to population stratification; we still need further evidence to assess the relationship in additional countries with different economic status and across additional ancestral backgrounds. Third, it is hard to reach the conclusion that simply increasing education would help people lower their risk of cardiovascular diseases, since little is known about the underlying mechanism of the genetic effects.

In conclusion, our MR results demonstrated the causal effect of longer education on the decreased odds of having cardiometabolic diseases. However, the findings may need further replications in other large, prospective studies.

## Data Availability Statement

Publicly available datasets were analyzed in this study. This data can be found here: the Genetic Investigation of ANthropometric Traits (GIANT) consortium, the Diabetes Genetics Replication and Meta-analysis (DIAGRAM) consortium, and the Coronary Artery Disease Genome-wide Replication and Meta-analysis (CARDIoGRAM) plus the Coronary Artery Disease (C4D) Genetics (CARDIoGRAMplusC4D) consortium.

## Ethics Statement

All data used are publicly available. Ethical review and informed consent had been obtained in all of the original studies.

## Author Contributions

BC and MC designed the study and revised the manuscript critically. MC analyzed data and wrote the draft of the manuscript. All authors gave final approval of the version to be published.

## Conflict of Interest

The authors declare that the research was conducted in the absence of any commercial or financial relationships that could be construed as a potential conflict of interest.
