# Supplementary material for: Association of Educational Attainment With Adiposity, Type 2 Diabetes, and Coronary Artery Diseases: A Mendelian Randomization Study
Source: Front Public Health. 2020 Apr 22;8:112. doi: 10.3389/fpubh.2020.00112 (PMC7189805; doi:10.3389/fpubh.2020.00112)
Supplement: Supplementary file 1 [file Data_Sheet_1.docx]

Supplementary Table 1. Genetic association of EA with T2D and CAD.

| SNPs | EA | | T2D | | CAD | |
| --- | --- | --- | --- | --- | --- | --- |
|  | Beta | SE | Beta | SE | Beta | SE |
| rs10061788 | 0.021 | 0.003 | -0.0098 | 0.017 | -0.01112 | 0.01289 |
| rs1008078 | 0.016 | 0.003 | -0.014 | 0.013 | -0.00272 | 0.009551 |
| rs1043209 | 0.016 | 0.003 | 0.026 | 0.013 | 0.02231 | 0.009761 |
| rs10496091 | -0.012 | 0.003 | -0.0057 | 0.014 | 0.01714 | 0.01131 |
| rs11191193 | 0.019 | 0.003 | -0.0057 | 0.013 | -0.00548 | 0.01036 |
| rs11210860 | 0.016 | 0.003 | -0.0067 | 0.013 | 0.006925 | 0.01024 |
| rs112634398 | 0.041 | 0.006 | -0.074 | 0.031 | -0.07121 | 0.02953 |
| rs113520408 | 0.013 | 0.003 | -0.003 | 0.014 | -0.01004 | 0.01176 |
| rs114598875 | -0.014 | 0.003 | 0.013 | 0.018 | 0.02545 | 0.01456 |
| rs11588857 | 0.022 | 0.003 | -0.027 | 0.015 | 0.01904 | 0.01098 |
| rs11689269 | 0.012 | 0.003 | -0.015 | 0.013 | -0.00033 | 0.009705 |
| rs11690172 | 0.012 | 0.003 | -0.015 | 0.012 | -0.0143 | 0.009828 |
| rs11712056 | -0.025 | 0.002 | 0.015 | 0.012 | 0.01971 | 0.009882 |
| rs11768238 | -0.014 | 0.003 | 0.0016 | 0.013 | 0.00828 | 0.009954 |
| rs12531458 | 0.011 | 0.002 | 0.0051 | 0.012 | -0.01239 | 0.009426 |
| rs12646808 | -0.013 | 0.003 | 0.028 | 0.013 | 0.01715 | 0.01024 |
| rs12671937 | 0.014 | 0.003 | -0.0074 | 0.012 | -0.01335 | 0.009782 |
| rs12682297 | -0.014 | 0.002 | -0.024 | 0.013 | 0.006151 | 0.009634 |
| rs12772375 | 0.016 | 0.003 | -0.026 | 0.013 | -0.01479 | 0.009615 |
| rs12969294 | -0.018 | 0.003 | 0.012 | 0.013 | -0.0089 | 0.009668 |
| rs12987662 | 0.022 | 0.003 | -0.028 | 0.012 | -0.01334 | 0.009922 |
| rs13294439 | -0.025 | 0.003 | -0.042 | 0.012 | 0.003039 | 0.009481 |
| rs1402025 | 0.018 | 0.003 | -0.038 | 0.015 | -0.02349 | 0.01126 |
| rs165633 | -0.012 | 0.003 | 0.045 | 0.014 | 0.01803 | 0.01158 |
| rs16845580 | -0.016 | 0.003 | -0.0063 | 0.013 | -0.00247 | 0.009797 |
| rs17119973 | -0.018 | 0.003 | 0.026 | 0.014 | 0.006276 | 0.01143 |
| rs17167170 | 0.017 | 0.003 | -0.016 | 0.015 | 0.000135 | 0.0124 |
| rs1777827 | -0.014 | 0.003 | -0.0035 | 0.013 | -0.01079 | 0.009734 |
| rs17824247 | 0.018 | 0.003 | -0.0048 | 0.012 | 0.002387 | 0.00992 |
| rs192818565 | -0.019 | 0.003 | 0.004 | 0.017 | 0.04197 | 0.01468 |
| rs2431108 | 0.012 | 0.003 | -0.017 | 0.013 | -0.00468 | 0.01016 |
| rs2456973 | 0.018 | 0.003 | -0.024 | 0.013 | 0.002763 | 0.01023 |
| rs2457660 | 0.016 | 0.003 | 0.02 | 0.013 | -0.02524 | 0.01034 |
| rs2568955 | 0.015 | 0.003 | 0.013 | 0.015 | -0.00686 | 0.01116 |
| rs2610986 | -0.01 | 0.003 | 0.033 | 0.014 | 0.02988 | 0.01112 |
| rs2615691 | 0.023 | 0.007 | -0.032 | 0.032 | -0.00769 | 0.02126 |
| rs2837992 | -0.013 | 0.003 | 0.016 | 0.013 | 0.01112 | 0.009938 |
| rs2964197 | 0.014 | 0.002 | -0.022 | 0.012 | -0.01586 | 0.009295 |
| rs2992632 | -0.017 | 0.003 | -0.0052 | 0.014 | 0.01138 | 0.01054 |
| rs301800 | -0.017 | 0.003 | 0.017 | 0.016 | 0.009171 | 0.01178 |
| rs3101246 | 0.008 | 0.003 | -0.0072 | 0.013 | 0.01098 | 0.009566 |
| rs34072092 | -0.016 | 0.004 | -0.038 | 0.021 | 0.008116 | 0.01629 |
| rs34305371 | 0.036 | 0.004 | 0.058 | 0.022 | 0.01557 | 0.01822 |
| rs35761247 | 0.034 | 0.006 | -0.0041 | 0.032 | -0.07989 | 0.02416 |
| rs4493682 | 0.019 | 0.003 | 0.024 | 0.016 | -0.01513 | 0.01183 |
| rs4863692 | -0.018 | 0.003 | -0.0023 | 0.013 | 0.006001 | 0.0098 |
| rs55830725 | -0.018 | 0.003 | 0.016 | 0.016 | 0.01756 | 0.01253 |
| rs56231335 | 0.016 | 0.003 | -0.026 | 0.013 | -0.00627 | 0.01056 |
| rs572016 | -0.011 | 0.002 | 0.035 | 0.012 | 0.02192 | 0.009141 |
| rs61160187 | -0.018 | 0.003 | 0.018 | 0.013 | -0.00485 | 0.01017 |
| rs62259535 | 0.032 | 0.008 | 0.072 | 0.035 | -0.03145 | 0.0351 |
| rs62263923 | -0.016 | 0.003 | 0.011 | 0.013 | 0.008655 | 0.009664 |
| rs62379838 | -0.012 | 0.003 | 0.016 | 0.013 | 0.01923 | 0.01029 |
| rs6739979 | 0.013 | 0.003 | -0.0031 | 0.012 | -0.00818 | 0.009649 |
| rs6799130 | -0.011 | 0.002 | 0.015 | 0.012 | -0.00826 | 0.009318 |
| rs7131944 | 0.013 | 0.003 | 0.0024 | 0.013 | -0.00236 | 0.009576 |
| rs7306755 | 0.025 | 0.003 | -0.052 | 0.015 | -0.0216 | 0.01192 |
| rs76076331 | -0.018 | 0.004 | 0.015 | 0.017 | 0.01157 | 0.01438 |
| rs7767938 | -0.013 | 0.003 | 0.037 | 0.014 | -0.00167 | 0.01057 |
| rs7854982 | 0.013 | 0.002 | -0.018 | 0.012 | 0.003941 | 0.009481 |
| rs7945718 | 0.014 | 0.003 | 0.0024 | 0.013 | -0.02184 | 0.009804 |
| rs7955289 | 0.016 | 0.003 | -0.0023 | 0.013 | -0.01085 | 0.009687 |
| rs8005528 | -0.017 | 0.004 | -0.0084 | 0.014 | 0.01861 | 0.01081 |
| rs895606 | -0.013 | 0.002 | -0.016 | 0.012 | 0.002629 | 0.009509 |
| rs9320913 | 0.024 | 0.002 | -0.024 | 0.012 | 0.000508 | 0.009408 |
| rs9537821 | 0.023 | 0.003 | -0.021 | 0.013 | 0.000516 | 0.011 |

Supplementary Table 2. Genetic association of EA with BMI.

| SNPs | EA | | BMI | |
| --- | --- | --- | --- | --- |
|  | Beta | SE | Beta | SE |
| rs1008078 | 0.016 | 0.003 | -0.0075 | 0.0038 |
| rs1043209 | 0.016 | 0.003 | -0.0023 | 0.0038 |
| rs10496091 | -0.012 | 0.003 | 0.0062 | 0.0034 |
| rs11191193 | 0.019 | 0.003 | -0.0014 | 0.004 |
| rs11210860 | 0.016 | 0.003 | -8.00E-04 | 0.0038 |
| rs113520408 | 0.013 | 0.003 | -0.0071 | 0.0046 |
| rs11588857 | 0.022 | 0.003 | 6.00E-04 | 0.0052 |
| rs11689269 | 0.012 | 0.003 | -0.0077 | 0.004 |
| rs11690172 | 0.012 | 0.003 | -0.009 | 0.0038 |
| rs11712056 | -0.025 | 0.002 | 0.0097 | 0.0038 |
| rs11768238 | -0.014 | 0.003 | 0.001 | 0.0033 |
| rs12531458 | 0.011 | 0.002 | -2.00E-04 | 0.0037 |
| rs12671937 | 0.014 | 0.003 | 0.0091 | 0.0031 |
| rs12969294 | -0.018 | 0.003 | 0.0044 | 0.004 |
| rs12987662 | 0.022 | 0.003 | -0.0112 | 0.0038 |
| rs13294439 | -0.025 | 0.003 | -0.0132 | 0.0528 |
| rs1402025 | 0.018 | 0.003 | -0.0082 | 0.0044 |
| rs16845580 | -0.016 | 0.003 | 0.0045 | 0.0038 |
| rs17119973 | -0.018 | 0.003 | 0.004 | 0.0043 |
| rs17167170 | 0.017 | 0.003 | -1.00E-04 | 0.0046 |
| rs1777827 | -0.014 | 0.003 | -0.0042 | 0.004 |
| rs192818565 | -0.019 | 0.003 | 0.0034 | 0.0047 |
| rs2456973 | 0.018 | 0.003 | -0.0084 | 0.004 |
| rs2457660 | 0.016 | 0.003 | -5.00E-04 | 0.0041 |
| rs2568955 | 0.015 | 0.003 | 0.0203 | 0.0046 |
| rs2615691 | 0.023 | 0.007 | 0.0063 | 0.0127 |
| rs2837992 | -0.013 | 0.003 | 0.0094 | 0.0038 |
| rs2964197 | 0.014 | 0.002 | -0.0025 | 0.0031 |
| rs2992632 | -0.017 | 0.003 | 0.0081 | 0.0042 |
| rs301800 | -0.017 | 0.003 | 0.0093 | 0.0049 |
| rs3101246 | 0.008 | 0.003 | 0 | 0.0033 |
| rs34072092 | -0.016 | 0.004 | 0.0031 | 0.0063 |
| rs34305371 | 0.036 | 0.004 | 0.0264 | 0.0055 |
| rs4493682 | 0.019 | 0.003 | 0.0031 | 0.0049 |
| rs4863692 | -0.018 | 0.003 | 0.0076 | 0.0041 |
| rs55830725 | -0.018 | 0.003 | 0.007 | 0.0051 |
| rs56231335 | 0.016 | 0.003 | -0.0159 | 0.0044 |
| rs572016 | -0.011 | 0.002 | 0.0045 | 0.0031 |
| rs61160187 | -0.018 | 0.003 | -0.004 | 0.004 |
| rs62263923 | -0.016 | 0.003 | 0.0151 | 0.0038 |
| rs62379838 | -0.012 | 0.003 | -0.0097 | 0.004 |
| rs6799130 | -0.011 | 0.002 | 0 | 0.0037 |
| rs7306755 | 0.025 | 0.003 | 0.0038 | 0.0047 |
| rs76076331 | -0.018 | 0.004 | 0.004 | 0.0054 |
| rs7767938 | -0.013 | 0.003 | 0.0104 | 0.0043 |
| rs7854982 | 0.013 | 0.002 | 0.0013 | 0.0038 |
| rs7945718 | 0.014 | 0.003 | -0.0044 | 0.0044 |
| rs7955289 | 0.016 | 0.003 | -0.0022 | 0.004 |
| rs895606 | -0.013 | 0.002 | -0.0025 | 0.0037 |
| rs9320913 | 0.024 | 0.002 | -0.0091 | 0.0037 |
| rs9537821 | 0.023 | 0.003 | -0.0082 | 0.0042 |
| rs6739979 | 0.013 | 0.003 | 0.002 | 0.004 |
| rs114598875 | -0.014 | 0.003 | 0.0069 | 0.0046 |
| rs12682297 | -0.014 | 0.002 | 2.00E-04 | 0.0043 |
| rs2431108 | 0.012 | 0.003 | -0.0044 | 0.004 |
| rs7131944 | 0.013 | 0.003 | -0.0019 | 0.004 |
